# Hip fracture risk prediction in older adults: Effects of sampling ratio and calibration in random survival forests

**DOI:** 10.1016/j.bonr.2026.101966

**Published:** 2026-10-28

**Authors:** Katsuyuki Chida, Ayaka Hananoe, Tetsuo Ishikawa, Eiryo Kawakami

**Affiliations:** a Department of Artificial Intelligence Medicine, Graduate School of Medicine, Chiba University, 1-8-1 Inohana, Chuo-ku, Chiba-shi, Chiba, 260-8670, Japan; b Predictive Medicine Special Project (PMSP), RIKEN Center for Integrative Medical Sciences (IMS), RIKEN, 1-7-22 Suehiro-cho, Tsurumi-ku, Yokohama, Kanagawa, 230-0045, Japan; c Division of Applied Mathematical Science, RIKEN Center for Interdisciplinary Theoretical and Mathematical Sciences (iTHEMS), RIKEN, 2-1 Hirosawa, Wako, Saitama, 351-0198, Japan; d Department of Extended Intelligence for Medicine, The Ishii-Ishibashi Laboratory, Keio University School of Medicine, 35 Shinanomachi, Shinjuku-ku, Tokyo, 160-8582, Japan; e Collective Intelligence Research Laboratory, Graduate School of Arts and Sciences, The University of Tokyo, 3-8-1 Komaba, Meguro-ku, Tokyo, 153-8902, Japan; f Advanced Artificial Intelligence Medicine, Graduate School of Medicine, The University of Osaka, 2-2 Yamada-oka, Suita, Osaka, 565-0871, Japan

**Keywords:** Hip fracture, Claims data, Health checkups, Random survival forest, Isotonic regression, Survival analysis, Model calibration

## Abstract

**Background:**

Hip fracture (HF) is common among older adults. Predicting HF risk may support preventive screening, but rare-event survival modeling presents calibration challenges. Building on prior evidence favoring random survival forests (RSF), we evaluated case-control sampling ratios and post-hoc calibration in RSF, benchmarked against standard Cox regression.

**Methods:**

In this retrospective claims-based cohort of 2,388,375 individuals (19,463 incident HFs), RSF and Cox models were trained across sampling ratios (1:1 to 1:50) with 100 ID-level bootstrap repetitions and post-hoc reweighted isotonic survival regression (RW-ISR). Performance over 6 years was evaluated among individuals aged ≥75 years using C-index and integrated Brier Skill Score (IBSS).

**Results:**

For HF prediction among individuals aged ≥75 years, RW-ISR consistently improved calibration while preserving discrimination. The highest median C-index was 0.770 for both RSF and clinical Cox, substantially exceeding demographic baselines (0.728). Calibration was highest under 1:1 sampling with RW-ISR, where clinical Cox achieved superior calibration skill compared with RSF (median IBSS: 0.214 vs. 0.159). Partial dependence in RSF showed clinically plausible associations.

**Conclusions:**

RW-ISR restored calibration without compromising discrimination. Notably, clinical Cox achieved discrimination comparable to RSF (median C-index 0.770 for both) and superior calibration, indicating that the more complex machine-learning model did not confer additional predictive benefit over standard regression in this older population. Across both models within the experimental conditions examined, optimal sampling ratios differed between discrimination and calibration objectives. Routine claims and checkup data enabled effective population-level risk stratification.

## Highlights

• Random survival forests predicted hip fracture from claims and checkup data.
• Isotonic regression substantially improved model calibration.
• The best sampling ratio for calibration differed from that for discrimination.
• Age, eGFR, BMI, and ALT showed plausible associations with hip fracture risk.
• The framework achieved reasonable prediction performance in older adults.

## Graphical abstract

**Figure:**
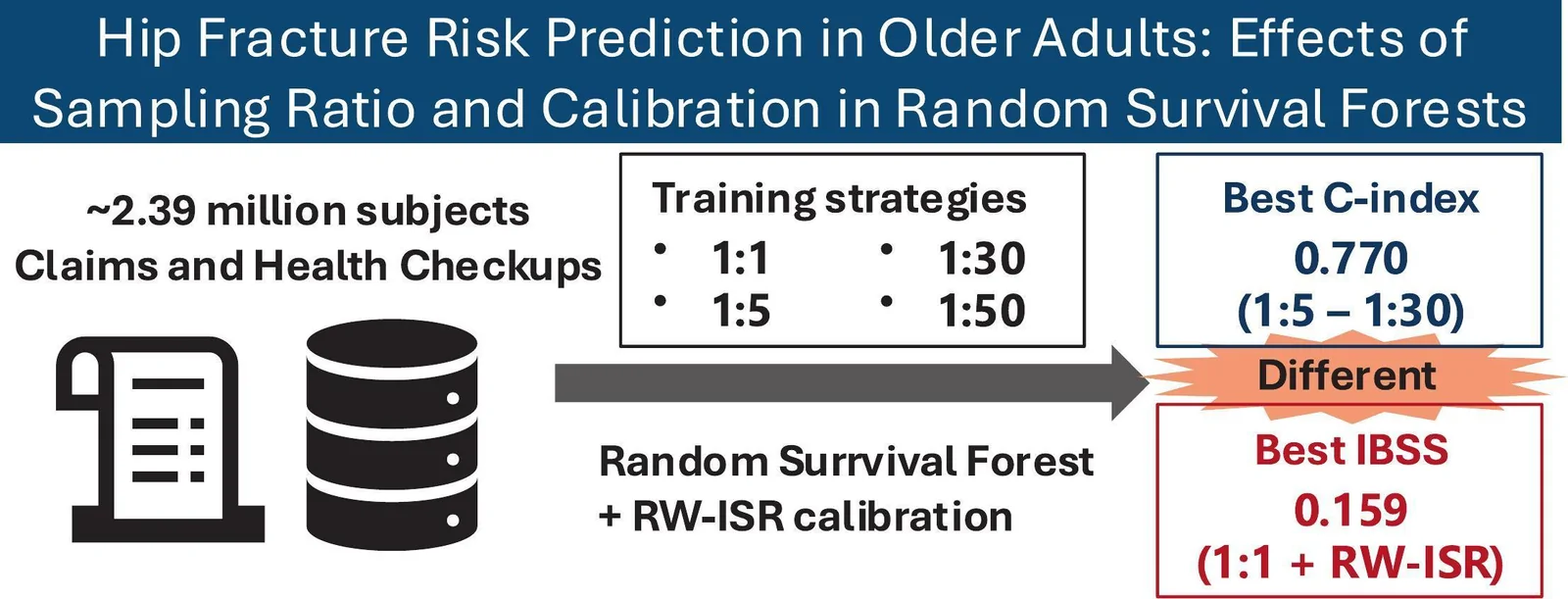

## 1 Introduction

Hip fracture (HF) is one of the most commonly seen pathological fractures, particularly in the elderly. Although incidence rates of HF have decreased in many countries, this decline is insufficient to offset the impact of population aging, resulting in an increase in the absolute number of patients in Japan and worldwide (Sing et al., 2023; Orimo et al., 2016). In Japan, the annual incidence of HF in 2017 reached 156.5 per 100,000 in males and 315.5 per 100,000 in females aged 70–79 years, with incidence increasing further at older ages (Takusari et al., 2021). HF increases the medical cost by three times (Haentjens et al., 2001) and decreases patients' quality of life (Cooper, 1997).

Early risk identification is important because the multifactorial interventions, such as hip protectors, exercise, and home safety assessment and modification, in the higher-risk population significantly reduce fractures or falls (Koike et al., 2009; Gillespie et al., 2012; Montero-Odasso et al., 2021). Some scoring methods, including FRAX, Garvan, and QFracture, were validated and used for the prediction of HF (Sun et al., 2022). In addition, recent studies applying artificial intelligence (AI) methods, such as XGBoost, have demonstrated improved predictive performance for fracture classification in older populations (Uragami et al., 2023; Engels et al., 2020). However, widely used tools such as FRAX are models for the clinical assessment of fracture probability (Kanis et al., 2008). In aging societies, where a large proportion of older adults are classified as being at elevated risk, this approach may result in a substantial number of individuals being flagged as candidates for intervention. It becomes difficult to prioritize limited resources efficiently based solely on absolute risk thresholds (Orimo et al., 2012).

Relative risk prediction AI models, such as random survival forests (RSF) (Ishwaran et al., 2008), enable more granular stratification of individuals according to their risk ranking within a population. Such approaches are particularly suited for population-level applications, including insurance programs and public health initiatives, where the goal is not only individual risk assessment but also the optimization of resource allocation and the reduction of healthcare costs. Our previous work suggested that RSF outperforms other models in relative risk prediction using claims and health checkup data (Kashiwagi et al., 2026). Because administrative databases routinely capture large populations without additional clinical examination burden but lack bone-specific measures such as bone mineral density (BMD), such population-level models are intended to complement rather than replace individualized clinical risk assessment.

Accurate risk ranking alone may be insufficient for many clinical and public health applications. Decisions regarding preventive interventions, resource allocation, and risk communication often require well-calibrated absolute risk estimates in addition to strong discrimination. Although RSF provides strong discriminative performance, the predicted survival probabilities may not always be well calibrated. Post-hoc calibration methods such as reweighted isotonic survival regression (RW-ISR) may address this limitation by improving agreement between predicted and observed risks while preserving the discriminatory ability of the underlying model (Jain et al., 2026).

Building upon previous evidence where RSF demonstrated superior discrimination (Kashiwagi et al., 2026), we evaluated an RSF-based survival prediction framework for incident HF events in older adults aged ≥75 years, with specific focus on how case-control sampling ratios interact with post-hoc calibration (RW-ISR). We rigorously benchmarked RSF against Cox proportional hazards models. We further assessed whether post-hoc calibration could enhance the reliability of absolute risk estimates while preserving the risk-stratification performance across multiple sampling ratios, and evaluated single-record sensitivity analyses to verify robustness against repeated observations.

## 2 Methods

### 2.1 Database

Data were obtained from the DeSC database provided by DeSC Healthcare, Inc., a large-scale Japanese healthcare database containing health insurance claims and annual health checkup records derived from multiple insurance schemes, including employees' health insurance societies, the National Health Insurance system, and the Late-stage Elderly Medical Care System. Individuals registered between April 2014 and February 2022 who had undergone at least one health checkup were eligible for inclusion. A total of 2,388,375 individuals were included in the analysis.

### 2.2 Study design

We employed fixed case-control sampling ratios and ID-level bootstrap resampling for model development (Fig. 1A). Individuals were classified as cases if they had a diagnosis of hip fracture (HF; International Statistical Classification of Diseases and Related Health Problems, 10th Revision [ICD-10], codes S720–S722) and as controls otherwise. A total of 19,463 cases and 2,368,912 controls were identified. Cases and controls were randomly divided into training, calibration, and evaluation subsets at a ratio of 8:1:1 at the individual level. The resulting training subset was used as a source population for subsequent bootstrap sampling. Independent calibration and evaluation datasets were established before bootstrap sampling and were shared across all sampling ratios and bootstrap iterations. Training datasets were generated from the training source population using fixed case-control sampling ratios of 1:1, 1:5, 1:30, and 1:50. To ensure comparable sample sizes across training conditions, each training dataset contained 50,000 records. For each sampling ratio, 100 ID-level bootstrap repetitions were performed.

**Fig. 1:**
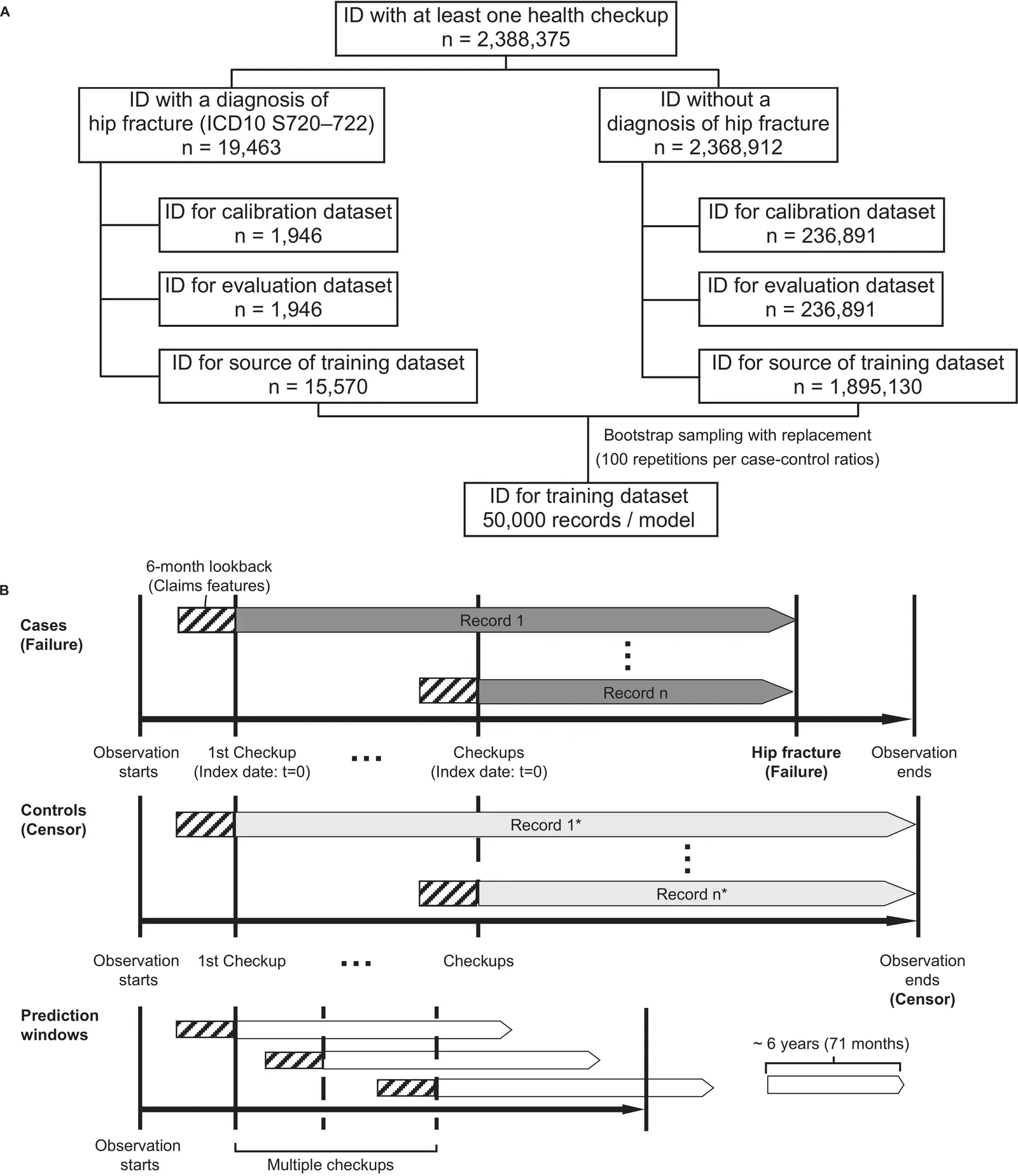
Study design and dataset construction. (A) Participant selection and creation of bootstrap training datasets. Independent calibration and evaluation datasets were established once and were reused across all bootstrap iterations and sampling ratios. Bootstrap training datasets were subsequently generated under multiple case-control sampling ratios. (B) Schematic representation of record construction and outcome definition. Health checkup records from the same individual could contribute multiple observations. Explanatory features were derived from the 6-month period preceding the health checkup (lookback window), while event follow-up started after the checkup date. Each record was linked to subsequent fracture occurrence or censoring and was treated as an independent observation during model training and evaluation.

As illustrated in Fig. 1B, each health checkup record was treated as an independent observation. Multiple records originating from the same individual could therefore contribute to the analysis. The date of each health checkup was defined as the index date (time = 0), and subsequent HF occurrence within the observable period was used as the prediction outcome. Records without subsequent HF diagnosis were treated as censored observations at the end of follow-up.

The primary clinical outcome was defined as the first recorded HF diagnosis (ICD-10: S720–S722) occurring strictly after the index health checkup date. Because hip fracture is an acute event, diagnoses recorded before the index checkup date were considered prior fractures rather than incident outcomes, and individuals with prior HF were excluded from the incident event definition. Prior validation studies in Japanese and East Asian claims databases have demonstrated high diagnostic accuracy for claims-based HF diagnosis codes (sensitivity 81.9% and specificity 99.2%) (Lee et al., 2022; Hudson et al., 2013). In accordance with the acute presentation of hip fractures, no arbitrary washout period was applied; instead, the entire observation period preceding each checkup was used to verify the absence of prior fracture.

In the primary analysis, annual health checkup records were evaluated as repeated screening observations. To confirm that within-individual record correlation did not introduce optimistic bias, we conducted two single-record sensitivity analyses: (1) using each individual's first eligible checkup record, and (2) using each individual's latest eligible checkup record prior to event or censoring. Importantly, the latest-checkup approach is designated strictly as a retrospective sensitivity analysis to evaluate potential selection bias and record-density effects, rather than as a prospective clinical prediction strategy since the latest pre-event record cannot be identified prospectively in clinical practice.

### 2.3 Feature extraction

Disease-related binary features were extracted from claims records. To reduce dimensionality and exclude rare diagnoses, only ICD-10 three-digit categories present in more than 1% of all individuals were retained, resulting in 394 diagnostic binary variables. For each health checkup record, diagnoses recorded during the preceding 6 months were aggregated and used as explanatory variables. Age at the index health checkup and sex were included as demographic features. In models developed using the entire population, insurer category was additionally included as a predictor. Because insurer category showed little variation among individuals aged ≥75 years, this variable was omitted from age-restricted models. Health checkup variables derived from routine examinations were harmonized across measurement methods and coding schemes. Original measurement variables with high missingness (≥50%) were excluded during preprocessing to reduce sparsity in the predictor set, whereas derived clinical variables generated from these measurements were retained because they integrated information across multiple measurement methods and provided more complete representations of the underlying clinical characteristics. A total of 46 checkup variables were included in the final analysis. Missing-value indicators were additionally generated for health checkup variables containing missing observations. In RSF models, missing values in continuous checkup features were handled using extremal imputation alongside explicit missing-value indicator variables. A comprehensive Feature Dictionary detailing all candidate predictor variables (394 ICD-10 diagnostic categories, 46 harmonized health checkup variables, missingness indicators, and demographics) is provided in Supplementary Table S1 and Supplementary Data 1.

### 2.4 Model development and calibration

RSF were implemented using 500 survival trees with a maximum tree depth of 15 and a minimum terminal node size of 250. Models were independently trained on each bootstrap dataset generated from the predefined case-control sampling ratios. To improve calibration of predicted survival probabilities, RW-ISR was applied as a post-hoc calibration step (Jain et al., 2026). The calibration dataset retained the original event prevalence of the target population and was used to adjust predicted survival probabilities to match observed event frequencies. Predicted survival probabilities from the RSF were obtained for the calibration dataset at monthly time points between 1 and 71 months after the index checkup. For each time point, isotonic regression was fitted using inverse probability of censoring weighting (IPCW) to account for right-censored observations. IPCW weights were estimated from the Kaplan–Meier estimator of the censoring distribution. In addition, Dykstra-based correction was evaluated to enforce monotonicity simultaneously across survival time and risk strata. The method iteratively applied isotonic projections in both dimensions using Dykstra's alternating projection algorithm (Bauschke and Borwein, 1994).

Four prediction strategies were evaluated: RSF (uncalibrated prediction), RSF-ISR (RW-ISR calibration), RSF-Dykstra, and RSF-ISR-Dykstra. For survival-function-based analyses, risk scores were calculated from the predicted survival function $\hat{S}\left(t\right)$ as

$$\mathrm{Risk}=\int_{0}^{\tau }-\log\left\{\hat{S}\left(t\right)\right\}\mathrm{dt}$$

where the integral was approximated numerically using the trapezoidal rule over monthly time points from 1 to 71 months.

To establish standard clinical reference baselines, three benchmark Cox proportional hazards models were implemented and evaluated under identical bootstrap sampling and calibration conditions: (1) Demographic Cox model, including age and sex; (2) Clinical Cox model, including age, sex, and all 46 routine health checkup variables; and (3) All-features Cox model, including all 488 candidate predictors. To ensure standardized comparisons and mitigate potential multicollinearity across varying feature dimensions, all benchmark Cox models were implemented as penalized Cox proportional hazards models with an elastic net penalty (mixing parameter $\ell_{1}\;\text{ratio}=0.5$) using CoxnetSurvivalAnalysis, with missing continuous values imputed using median imputation and predictors standardized prior to model fitting.

### 2.5 Model evaluation

Two prediction strategies were evaluated for RSF and RSF-ISR models. The first strategy trained RSF models using only individuals aged ≥75 years. The second strategy trained RSF models using all eligible individuals and subsequently recalibrated survival probabilities using the calibration dataset restricted to individuals aged ≥75 years. Recalibration was performed by refitting the RW-ISR models using the age-restricted calibration dataset while preserving the trained RSF model.

Model discrimination was assessed using the concordance index (C-index). Calibration performance was evaluated using the integrated Brier Skill Score (IBSS), defined as

$$\mathrm{IBSS}=1-\frac{\mathrm{IB}S_{\mathrm{model}}}{\mathrm{IB}S_{\mathrm{KM}}}$$

where $\mathrm{IB}S_{\mathrm{model}}$ and $\mathrm{IB}S_{\mathrm{KM}}$ denote integrated Brier score of evaluated model and the Kaplan–Meier reference model, respectively. The integrated Brier score was calculated using inverse probability of censoring weighting (Graf et al., 1999). Higher IBSS values indicate better calibration performance relative to the Kaplan–Meier estimator. Both metrics were evaluated using the independent evaluation dataset.

In addition to global IBSS and C-index, multi-horizon calibration was evaluated at 1-year (12 months), 3-year (36 months), and 5-year (60 months) follow-up points using calibration slope and calibration intercept, estimated via logistic calibration on IPCW-weighted observed outcomes. Both a calibration slope of 1.0 and an intercept of 0 represent ideal calibration.

To quantify uncertainty associated with training-data sampling, model development and evaluation were repeated across 100 ID-level bootstrap samples for each case-control sampling ratio. Median performance, interquartile ranges, and 95% bootstrap confidence intervals were reported.

Model interpretation was performed using partial dependence plots (PDPs) for age, estimated glomerular filtration rate (eGFR), body mass index (BMI), and alanine aminotransferase (ALT). PDPs were generated for each bootstrap model individually by replacing the feature of interest with predefined grid values while keeping all other variables unchanged. For each grid value, risk scores were computed from the predicted survival function and averaged across all observations. Mean PDPs and 95% bootstrap intervals were subsequently calculated from the empirical distribution of the resulting PDP estimates.

### 2.6 Statistical software

All analyses were conducted using Python version 3.14.2. RSF and Cox proportional hazards models were implemented using the scikit-survival package (version 0.27.0), and calibration procedures were implemented using scikit-learn (version 1.8.0).

### 2.7 Reporting and ethical considerations

This study was reported in accordance with the TRIPOD+AI (Transparent Reporting of a multivariable prediction model for Individual Prognosis Or Diagnosis + Artificial Intelligence) statement (Collins et al., 2024); the completed checklist is provided as Supplementary Material. The Research Ethics Committee of the Graduate School of Medicine, Chiba University, determined that ethical review was not required because this study used only fully anonymized data that did not constitute personal information. The requirement for individual informed consent was also waived because the study used anonymized, retrospectively collected administrative claims and health checkup data, in accordance with the Ethical Guidelines for Medical and Biological Research Involving Human Subjects issued by the Japanese government. All procedures were performed in accordance with the Declaration of Helsinki.

## 3 Results

### 3.1 Characteristics of the analysis population

Baseline characteristics are shown in Table 1. In all datasets, hip fracture cases were older and had a higher proportion of females than controls, consistent with previous reports. Cases also tended to have shorter observation periods and fewer health checkup records. Most characteristics differed significantly between groups. Among individuals aged ≥75 years, the event proportion of HF was 2.49% (14,143 of 568,980 individuals). At the record level (evaluating dynamic annual health checkup observations, distinct from unique individual counts), the independent evaluation dataset restricted to individuals aged ≥75 years comprised 106,572 health checkup records containing 1516 incident hip fracture events (1.42%), with the remaining 105,056 records representing right-censored observations.

**Table 1:** Characteristics of the training, calibration, and evaluation datasets.

Group	Characteristic^1^	Case N = 19,463	Control N = 2,368,912	*p*-Value^2^
Training		N = 15,570	N = 1,895,130	
	Sex			<0.0001
	Male	3504 (23%)	892,538 (47%)	
	Female	12,066 (77%)	1,002,592 (53%)	
	Age at observation start, years	79 (70–85)	64 (51–71)	<0.0001
	Observation period, months	37 (35–71)	50 (35–71)	<0.0001
	Months until first health checkup	7 (3–15)	7 (4–15)	0.61
	Number of health checkups	2 (1–3)	3 (1–4)	<0.0001
Calibration		N = 1946	N = 236,891	
	Sex			<0.0001
	Male	425 (22%)	111,464 (47%)	
	Female	1521 (78%)	125,427 (53%)	
	Age at observation start, years	79 (69–85)	64 (51–71)	<0.0001
	Observation period, months	38 (35–71)	49 (35–71)	0.0020
	Months until first health checkup	7 (3–15)	7 (4–15)	0.0064
	Number of health checkups	2 (1–3)	3 (1–4)	<0.0001
Evaluation		N = 1947	N = 236,891	
	Sex			<0.0001
	Male	432 (22%)	112,009 (47%)	
	Female	1515 (78%)	124,882 (53%)	
	Age at observation start, years	79 (69–85)	64 (51–71)	<0.0001
	Observation period, months	37 (35–71)	50 (35–71)	0.025
	Months until first health checkup	7 (3–15)	7 (4–15)	0.32
	Number of health checkups	2 (1–3)	3 (1–4)	<0.0001

1 Median (IQR) for continuous variables, n (%) for categorical variables.

2 Pearson's Chi-squared test; Wilcoxon rank sum test.

### 3.2 Performance of models trained under different case-control sampling ratios and calibration strategies

We compared the discriminative performance (C-index) and calibration performance (IBSS) of models trained using fixed case-control sampling ratios and subsequently calibrated using datasets retaining the original event prevalence (Table 2, Supplementary Tables S2 and S3, Fig. 2). Discriminative performance was largely unaffected by post-hoc calibration (Fig. 2A). Among individuals aged ≥75 years, models trained using sampling ratios closer to the observed population incidence generally achieved higher C-index values, with the best-performing models achieving a median C-index of 0.770 (Supplementary Table S2). In contrast, RW-ISR calibrated models consistently improved calibration performance as measured by IBSS, whereas Dykstra-based correction provided limited benefit (Fig. 2B, C). The highest median IBSS was achieved by the 1:1 RSF-ISR model (0.159, 95% bootstrap confidence interval 0.148–0.167).

**Table 2:** Summary of best-performing models for discrimination and calibration among individuals aged ≥75 years.

Performance objective	Model architecture	Training ratio	Median C-index (95% CI)	Median IBSS (95% CI)	5-Year calibration slope (95% CI)
**Optimal discrimination**	**RSF-ISR**	**1:30**	**0.770 (0.766–0.773)**	0.038 (0.032–0.046)	0.72 (0.66–0.78)
	RSF (Uncalibrated)	1:30	0.770 (0.767–0.773)	−0.190 (−0.225 to −0.155)	0.28 (0.24–0.32)
	Clinical Cox-ISR (Benchmark)	1:30	0.770 (0.766–0.775)	0.047 (0.042–0.052)	0.78 (0.71–0.85)
**Optimal calibration**	**RSF-ISR**	**1:1**	0.706 (0.701–0.711)	**0.159 (0.148–0.167)**	**0.75 (0.65–0.84)**
	Clinical Cox-ISR (Benchmark)	1:1	0.674 (0.670–0.678)	0.214 (0.208–0.219)	0.84 (0.77–0.91)
**Population prevalence**	RSF-ISR	1:50	0.769 (0.765–0.772)	0.037 (0.030–0.045)	0.69 (0.61–0.76)
	Clinical Cox-ISR	1:50	0.769 (0.762–0.774)	0.042 (0.035–0.052)	0.74 (0.66–0.81)
**Baseline benchmark**	Demographic Cox (age, sex)	1:30	0.728 (0.727–0.728)	−0.235 (−0.267 to −0.194)	0.35 (0.31–0.40)
	Demographic Cox (age, sex)	1:1	0.707 (0.707–0.707)	−14.947 (−15.137 to −14.739)	0.08 (0.06–0.10)

All models were evaluated on the independent evaluation dataset restricted to individuals aged ≥75 years across 100 ID-level bootstrap iterations. Values represent median (95% bootstrap confidence interval). Bold text denotes best-performing models under specified performance objectives. CI: confidence interval; IBSS: integrated Brier skill score; RSF: random survival forest; RW-ISR: reweighted isotonic survival regression.

**Fig. 2:**
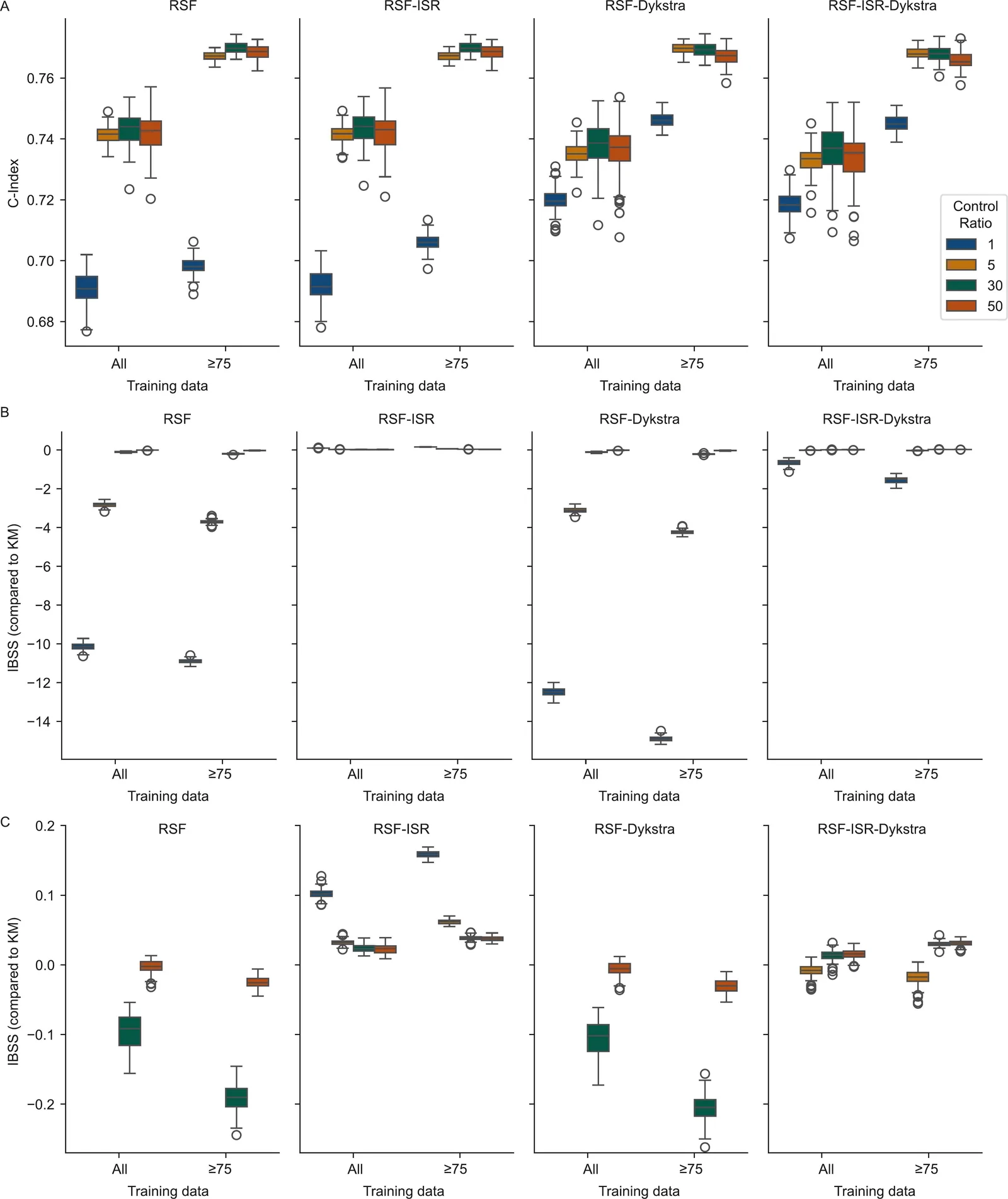
Predictive performance of RSF-based survival models trained under different case-control sampling ratios. (A) C-index. (B) Integrated Brier Skill Score (IBSS). (C) Enlarged view of panel B. Colors indicate the case-control sampling ratio used for model development (1:1, 1:5, 1:30, and 1:50). Boxplots represent the distribution of performance metrics across 100 ID-level bootstrap repetitions. Boxes represent the interquartile range (IQR), center lines indicate the median, whiskers extend to 1.5 × IQR, and circles represent outliers.

To benchmark RSF against standard survival regression, we evaluated three Cox proportional hazards models under identical bootstrap conditions (Supplementary Table S4). A demographic Cox model using only age and sex achieved a median C-index of 0.728 (95% CI 0.727–0.728). Adding the 46 routine health checkup variables (clinical Cox model) substantially improved discrimination, reaching a median C-index of 0.770 (95% CI 0.766–0.775) at a 1:30 ratio and matching the discriminative performance of RSF (0.770, 95% CI 0.766–0.773). A comprehensive Cox model incorporating all 488 features achieved a median C-index of 0.764 (95% CI 0.754–0.772). In terms of calibration, uncalibrated models trained on sampled data suffered from severe probability distortion; post-hoc RW-ISR substantially restored calibration across models, with 1:1 balanced sampling achieving the highest calibration for both RSF-ISR (median IBSS: 0.159, 95% CI 0.148–0.167) and the benchmark clinical Cox-ISR (median IBSS: 0.214, 95% CI 0.208–0.219; Table 2, Supplementary Table S4).

Horizon-specific calibration analysis at 1, 3, and 5 years demonstrated that post-hoc RW-ISR produced well-calibrated absolute risk estimates (Supplementary Table S5, Supplementary Fig. S1). For the 1:1 RSF-ISR model, calibration slopes were 0.91 (95% CI 0.86–0.95) at 1 year, 0.90 (95% CI 0.87–0.92) at 3 years, and 0.75 (95% CI 0.65–0.84) at 5 years, with calibration-in-the-large intercepts near zero (−0.04 to −0.12).

In single-record sensitivity analyses restricted strictly to one checkup record per individual, RSF models maintained consistent discrimination (Supplementary Table S6). When restricted to the first eligible checkup, the median C-index was 0.753 (95% CI 0.738–0.768) at 1:30 and 1:1 RSF-ISR achieved an IBSS of 0.147. In the retrospective sensitivity analysis restricted to the latest checkup prior to event or censoring, the median C-index was 0.767 (95% CI 0.752–0.781) and IBSS was 0.154. These findings demonstrate that model discrimination is robust and not an artifact of repeated observations.

Evaluating temporal monotonicity across the 71 monthly follow-up points showed that 96.1% of individual survival curves produced by independent RW-ISR were strictly monotonic, with local non-monotonic reversals occurring in 3.9% of curves (mean maximum probability increase of 0.089; Supplementary Table S7). While infrequent, such reversals indicate that independent point-wise recalibration does not guarantee temporal consistency across all individual survival trajectories.

### 3.3 Partial dependence on explanatory variables

Partial dependence analysis demonstrated clinically plausible associations between several key predictors and HF risk. Increasing age and decreasing eGFR were consistently associated with higher predicted fracture risk across sampling ratios and modeling strategies (Fig. 3A, B). Lower BMI and lower ALT levels were also associated with increased predicted HF risk in the present cohort (Fig. 3C, D). These associations were generally consistent with previously reported risk factors for osteoporotic fractures and frailty-related conditions (Irina et al., 2018; Vespasiani-Gentilucci et al., 2018; Harvey et al., 2025; Hsu et al., 2024). These curves reflect non-linear model-based risk profiles and should be interpreted as descriptive associations rather than causal relationships.

**Fig. 3:**
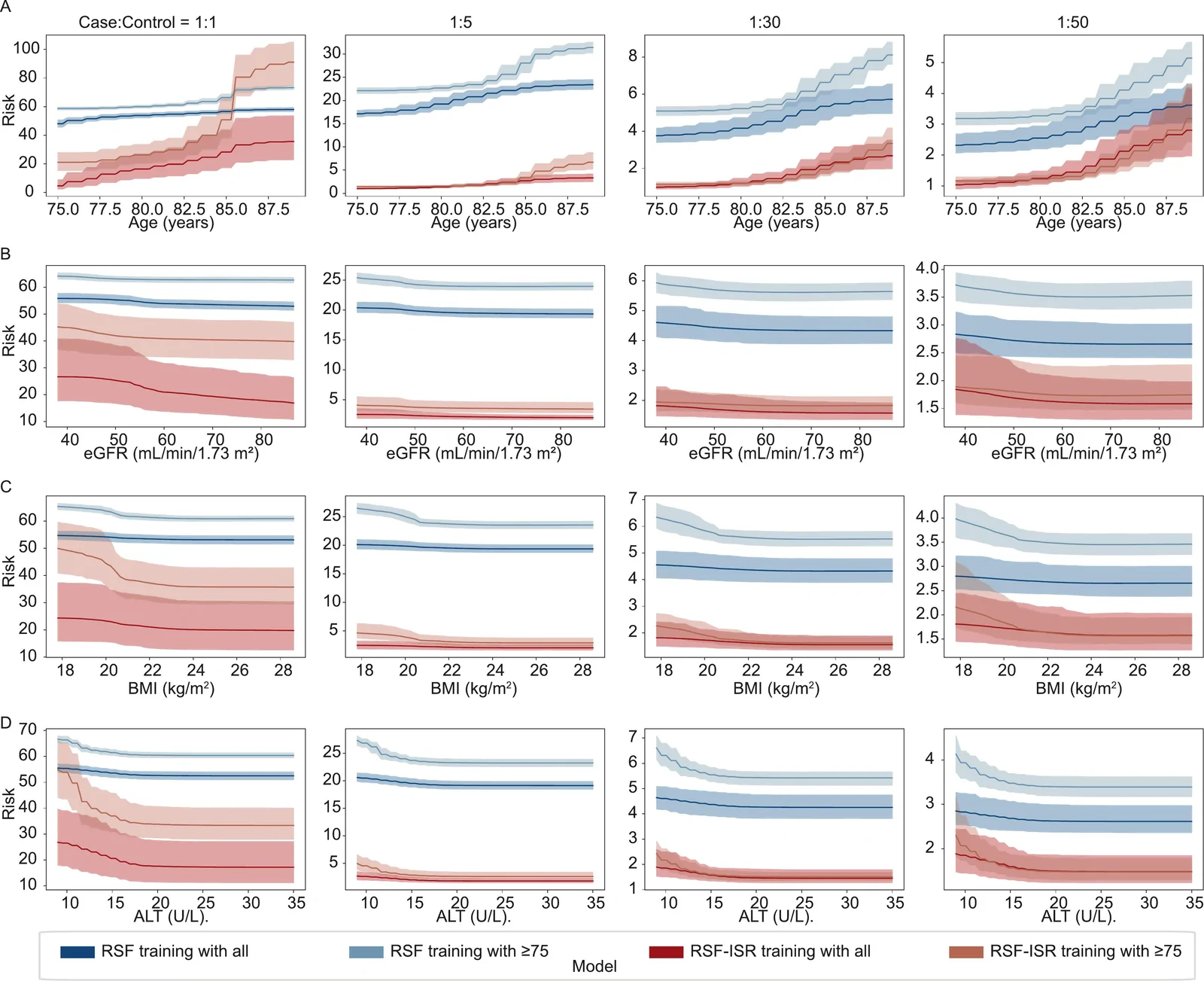
Partial dependence plots of selected predictors across different case-control sampling ratios. Partial dependence plots were generated for age (A), eGFR (B), BMI (C), and ALT (D). Columns represent case-control sampling ratios of 1:1, 1:5, 1:30, and 1:50. Dark and light blue curves indicate RSF models trained using all eligible individuals and participants aged ≥75 years, respectively. Dark and light red curves indicate RW-ISR calibrated models (RSF-ISR) trained using all eligible individuals and participants aged ≥75 years, respectively. Solid lines show the mean partial dependence across 100 bootstrap models and shaded areas indicate the 95% bootstrap interval. (For interpretation of the references to color in this figure legend, the reader is referred to the web version of this article.)

## 4 Discussion

The present study demonstrated that post-hoc RW-ISR improved the calibration performance of RSF and Cox models trained under varying event sampling ratios while preserving discriminative performance. Because RW-ISR recalibrates survival probabilities through a monotonic transformation that accounts for censoring without changing the rank ordering of individuals, it may provide a practical approach for adapting RSF-based risk predictions to target populations without retraining the underlying model.

Despite the challenges associated with predicting rare outcomes among older adults, the proposed framework achieved reasonable discrimination and calibration using only routinely collected claims and health checkup data. The ability to identify high-risk individuals and estimate their absolute risk using routinely collected healthcare data is particularly valuable in rapidly aging societies, where preventive resources are limited and efficient prioritization is required. Our results suggest that meaningful risk stratification can be achieved even within elderly populations, despite the relatively low incidence of HF events. Evaluating benchmark Cox proportional hazards models alongside RSF demonstrated that incorporating health checkup variables substantially improved discrimination over demographic predictors alone (C-index 0.770 vs. 0.728). Interestingly, in contrast to our previous findings in broader populations where RSF outperformed conventional models (Kashiwagi et al., 2026), the clinical Cox model here matched RSF in discrimination (C-index 0.770) and demonstrated superior calibration skill (median IBSS 0.214 vs. 0.159 under 1:1 sampling). This suggests that in older populations aged ≥75 years, quantitative continuous variables from routine checkups (e.g., eGFR, BMI, ALT) carry predominantly log-linear hazard signals that a well-specified clinical Cox model can capture effectively without requiring complex non-linear feature interactions, while sparse claims diagnosis codes provided minimal additional discrimination. Crucially, the fundamental dissociation between sampling ratios for discrimination (optimal at 1:30) and calibration (optimal at 1:1) was identically observed in benchmark Cox models (Supplementary Table S4), demonstrating that this trade-off was shared across both model classes within the experimental conditions investigated, rather than being an artifact specific to tree-based models. Furthermore, retrospective single-record sensitivity analyses confirmed that this discriminative ability was consistent (median C-index of 0.753 for the first checkup and 0.767 for the latest checkup) and not driven by repeated observations, though the latest-checkup analysis served strictly as a retrospective evaluation rather than a prospective tool.

Although the magnitude of the partial dependence profiles varied across sampling ratios, the overall directions of the associations were largely consistent with established risk factors for HF. Advanced age, lower eGFR, and lower BMI were associated with higher predicted risk, consistent with previous epidemiological studies (Harvey et al., 2025; Hsu et al., 2024). Lower ALT levels were also associated with increased predicted risk, which may reflect the reported relationship between low ALT activity, frailty, sarcopenia, and adverse outcomes in older adults (Irina et al., 2018; Vespasiani-Gentilucci et al., 2018). Collectively, these findings suggest that the models learned clinically plausible predictor-risk relationships despite differences in sampling strategy.

We originally hypothesized that differences between sampling strategies would primarily affect the scale of the predicted risks. However, partial dependence profiles after isotonic calibration showed that isotonic regression did not fully reconcile differences in predictor-response relationships observed across sampling strategies, which suggests that event sampling ratios may influence the feature-response relationships learned by RSF. Interestingly, models trained using a 1:1 event ratio showed the best calibration performance after isotonic regression despite not achieving the highest discrimination (Fig. 2). Models trained using 1:5 and 1:30 event ratios achieved the highest C-index values, whereas models trained using a 1:1 ratio exhibited the best calibration performance. These findings suggest that sampling ratios affect not only prediction bias but also the underlying risk representation learned by the model. In contrast to the common assumption that a single sampling ratio optimizes overall model performance, within the models and experimental conditions evaluated here, the optimal sampling ratios for discrimination did not coincide with that for calibration in rare-event survival. These findings suggest that sampling ratios may need to be determined separately for discrimination and calibration objectives depending on the clinical use case. Uncalibrated models trained under artificial sampling ratios exhibited negative IBSS values, reflecting severe probability distortion where uncalibrated predictions perform worse than the naive population Kaplan–Meier benchmark. Post-hoc RW-ISR successfully restored positive skill scores (IBSS 0.159) and achieved well-aligned calibration slopes across horizons (0.91 at 1 year, 0.90 at 3 years, and 0.75 at 5 years), demonstrating the necessity of recalibration when training on rebalanced datasets.

This study has several limitations. First, the claims database was originally collected for health insurance reimbursement purposes. Therefore, some recorded diagnoses may not necessarily represent confirmed clinical conditions. Although the HF diagnosis codes (ICD-10: S720–S722) have demonstrated relatively high sensitivity and specificity (81.9% and 99.2%, respectively) in previous studies (Lee et al., 2022; Hudson et al., 2013), the validity of predictor variables derived from claims-based diagnoses may be limited. Furthermore, claims and checkup records lack important clinical determinants such as bone mineral density (BMD), prior morphometric vertebral fractures, and falls history. Second, in our analysis, enforcing strict two-dimensional monotonicity constraints degraded calibration performance. Therefore, we adopted an independent timewise RW-ISR approach. Although local non-monotonic reversals occurred in only 3.9% of curves with modest magnitudes (mean increase 0.089), such violations should not be described as negligible without qualification, as they may affect individual risk estimates over time. Temporally consistent survival predictions remain important for future clinical applications. Third, model interpretation was based on PDP, which may evaluate unrealistic combinations of correlated predictors that are not represented in the underlying population. Therefore, the observed predictor-response profiles should be interpreted with caution. Fourth, only four case-control sampling ratios and two modeling frameworks were evaluated within a single healthcare database. The optimal balance between discrimination and calibration may differ according to outcome incidence, population characteristics, and model architecture, and our conclusions regarding sampling ratio divergence should be interpreted within these specific experimental boundaries. Fifth, while single-record sensitivity analyses supported the robustness of model discrimination, the primary model utilized repeated health checkup records without formal longitudinal trajectory modeling; furthermore, using the latest checkup was strictly a retrospective sensitivity analysis rather than a viable prospective prediction approach. Sixth, in this cohort of older adults aged ≥75 years, competing risks of non-fracture mortality were not explicitly modeled, which could lead to overestimation of absolute fracture incidence over extended follow-up horizons. Finally, this was a retrospective exploratory study conducted within a single Japanese claims database without external validation. The generalizability of our findings and the transportability of the proposed calibration framework must be rigorously evaluated in independent populations and international cohorts prior to clinical implementation.

## 5 Conclusion

Post-hoc RW-ISR calibration substantially improved absolute risk calibration in rare-event survival prediction while preserving discrimination. Notably, the standard clinical Cox proportional hazards model achieved discrimination comparable to RSF (C-index 0.770 for both) and demonstrated superior calibration performance (median IBSS 0.214 vs. 0.159 under 1:1 sampling), indicating that the additional complexity of RSF did not confer predictive advantages over a well-specified regression model in this older cohort. Furthermore, a shared dissociation between optimal sampling ratios for discrimination and calibration was observed across both modeling approaches under the investigated experimental conditions. These findings demonstrate that sampling strategies and recalibration must be aligned with specific clinical objectives, and that routinely collected claims and health checkup data can support effective population-level risk stratification rather than individual clinical decision-making. Future studies should evaluate the external validity and transportability of this framework in independent populations prior to clinical implementation.

## CRediT authorship contribution statement

**Katsuyuki Chida:** Writing – review & editing, Writing – original draft, Visualization, Validation, Methodology, Investigation, Funding acquisition, Formal analysis, Data curation. **Ayaka Hananoe:** Writing – review & editing, Visualization, Software, Formal analysis, Data curation. **Tetsuo Ishikawa:** Writing – review & editing, Validation, Methodology, Funding acquisition, Formal analysis. **Eiryo Kawakami:** Writing – review & editing, Writing – original draft, Visualization, Validation, Supervision, Resources, Project administration, Methodology, Funding acquisition, Conceptualization.

## Ethics declaration

The authors have NOT obtained informed consent from participants or their legal representatives. The requirement for individual informed consent was also waived because the study used anonymized, retrospectively collected administrative claims and health checkup data.

This study was performed in compliance with relevant laws, regulatory frameworks and guidelines where the research took place. This study was conducted in accordance with Ethical Guidelines for Medical and Biological Research Involving Human Subjects. Ethics committee approval was not required under relevant laws and institutional guidelines. This study used only fully anonymized data that did not constitute personal information.

## Declaration of competing interest

The authors declare the following competing interests: this study was conducted under a joint research agreement with DeSC Healthcare, Inc., which provided access to the DeSC database. DeSC Healthcare, Inc. had no role in the study design, analysis, interpretation of the data, or the decision to submit the manuscript for publication. The authors have no other competing financial interests or personal relationships that could have appeared to influence the work reported in this paper.

## Data Availability

The DeSC database, which includes claims data collected during routine medical practice, is not publicly available. Access to the dataset requires a Data Sharing Agreement with DeSC Healthcare, Inc. However, data required to reproduce the analysis may be shared upon reasonable request and with the permission of DeSC Healthcare, Inc. The source code is available at https://github.com/CHIDAKA/HipFracture_PredictionCalibration.
