# Supplementary material for: Hip fracture risk prediction in older adults: Effects of sampling ratio and calibration in random survival forests
Source: Bone Rep. 2026 Oct 28;31:101966. doi: 10.1016/j.bonr.2026.101966 (PMC13753595; doi:10.1016/j.bonr.2026.101966)
Supplement: Supplementary file 1 [file mmc1.pdf]

## Supplementary Figure and Tables

**Supplementary Figure S1.** Multi-horizon risk-decile calibration plots at 1-, 3-, and 5-year follow-up for uncalibrated RSF and calibrated RSF-ISR models (aged ≥75 years, 1:1 sampling ratio).

Blue squares indicate uncalibrated RSF predictions (displaying substantial risk overestimation due to the artificial 1:1 sampling prevalence). Red circles indicate post-hoc RW-ISR calibrated predictions, demonstrating close alignment with the ideal 45-degree dashed diagonal across all risk deciles

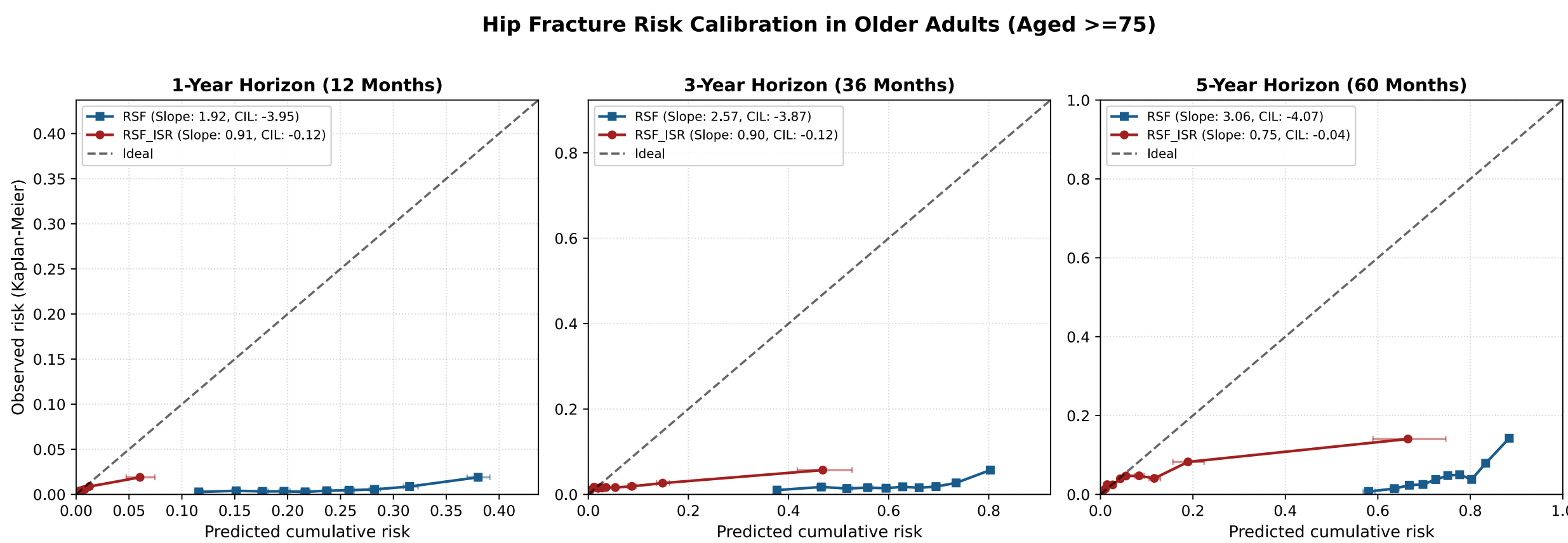

**Supplementary Table S1. Summary of Feature Dictionary.**

| Feature Domain | Variable Count | Description and Data Source | Measurement / Aggregation Window | Missing Data Handling |
|---|---|---|---|---|
| **Demographics** | 2 | Age at index checkup date (years); Sex (male/female). | Determined on the index checkup date. | No missing values permitted. |
| **ICD-10 Diagnoses** | 394 | Three-digit ICD-10 diagnostic categories with >1% prevalence. Binary indicators (1=present, 0=absent). | Derived from claims records during the 6-month lookback window preceding checkup date. | Zero-filled (absence of recorded diagnosis code). |
| **Routine Health Checkups** | 46 | Harmonized physical measurements, physiological parameters, and laboratory blood/urine test results. | Measured on the index health checkup date. | Extremal imputation with explicit missingness indicators. |
| **Missingness Indicators** | 46 | Binary indicator variables (1=missing, 0=observed) corresponding to each routine health checkup feature. | Generated during preprocessing on the index checkup date. | |

*Note:* Comprehensive feature manifest in Supplementary Data 1. ICD-10: International Classification of Diseases, Tenth Revision; RSF: random survival forest.

**Supplementary Table S2. C-index for each sampling ratio and model.**

| Model (Calibration) | Training data | Case-control Ratio (control / case) | Median | IQR | 95% bootstrap CI |
|---|---|---|---|---|---|
| **RSF** | All | 1 | 0.691 | 0.688 – 0.695 | 0.680 – 0.701 |
| | | 5 | 0.742 | 0.739 – 0.743 | 0.735 – 0.746 |
| | | 30 | 0.744 | 0.740 – 0.747 | 0.733 – 0.752 |
| | | 50 | 0.743 | 0.738 – 0.746 | 0.728 – 0.753 |
| | ≥75 | 1 | 0.698 | 0.697 – 0.700 | 0.693 – 0.704 |
| | | 5 | 0.767 | 0.766 – 0.768 | 0.765 – 0.770 |
| | | 30 | 0.770 | 0.768 – 0.771 | 0.767 – 0.773 |
| | | 50 | 0.769 | 0.767 – 0.770 | 0.765 – 0.772 |
| **RSF-ISR** | All | 1 | 0.691 | 0.689 – 0.696 | 0.681 – 0.702 |
| | | 5 | 0.742 | 0.740 – 0.743 | 0.735 – 0.747 |
| | | 30 | 0.744 | 0.740 – 0.747 | 0.733 – 0.753 |
| | | 50 | 0.742 | 0.738 – 0.746 | 0.728 – 0.753 |
| | ≥75 | 1 | 0.706 | 0.704 – 0.708 | 0.701 – 0.711 |
| | | 5 | 0.767 | 0.766 – 0.768 | 0.765 – 0.770 |
| | | 30 | 0.770 | 0.769 – 0.771 | 0.766 – 0.773 |
| | | 50 | 0.769 | 0.767 – 0.770 | 0.765 – 0.772 |
| **RSF-Dykstra** | All | 1 | 0.693 | 0.691 – 0.698 | 0.683 – 0.704 |
| | | 5 | 0.742 | 0.740 – 0.743 | 0.735 – 0.746 |
| | | 30 | 0.743 | 0.740 – 0.747 | 0.733 – 0.752 |
| | | 50 | 0.742 | 0.738 – 0.745 | 0.728 – 0.752 |
| | ≥75 | 1 | 0.750 | 0.748 – 0.752 | 0.746 – 0.754 |
| | | 5 | 0.767 | 0.766 – 0.768 | 0.764 – 0.770 |
| | | 30 | 0.770 | 0.768 – 0.771 | 0.766 – 0.773 |
| | | 50 | 0.769 | 0.767 – 0.770 | 0.765 – 0.772 |
| **RSF-ISR-Dykstra** | All | 1 | 0.693 | 0.690 – 0.697 | 0.683 – 0.704 |

| Model (Calibration) | Training data | Case-control Ratio (control / case) | Median | IQR | 95% bootstrap CI |
|---|---|---|---|---|---|
| | | 5 | 0.742 | 0.740 – 0.743 | 0.735 – 0.747 |
| | | 30 | 0.743 | 0.740 – 0.747 | 0.733 – 0.752 |
| | | 50 | 0.742 | 0.738 – 0.746 | 0.728 – 0.752 |
| | ≥75 | 1 | 0.749 | 0.747 – 0.750 | 0.746 – 0.753 |
| | | 5 | 0.767 | 0.766 – 0.768 | 0.764 – 0.770 |
| | | 30 | 0.770 | 0.768 – 0.771 | 0.766 – 0.773 |
| | | 50 | 0.769 | 0.767 – 0.770 | 0.765 – 0.772 |

*Note:* Values represent median, interquartile range (IQR, 25th–75th percentiles), and 95% bootstrap confidence interval across 100 ID-level bootstrap iterations. CI: confidence interval; IQR: interquartile range; RSF: random survival forest; RW-ISR: reweighted isotonic survival regression.

**Supplementary Table S3. Integrated Brier skill scores for each sampling ratio and model.**

| Model (Calibration) | Training data | Case-control Ratio (control / case) | Median | IQR | 95% bootstrap CI |
|---|---|---|---|---|---|
| **RSF** | All | 1 | -10.158 | -10.3 – -10.0 | -10.5 – -9.75 |
| | | 5 | -2.817 | -2.90 – -2.75 | -3.06 – -2.61 |
| | | 30 | -0.091 | -0.116 – -0.075 | -0.142 – -0.057 |
| | | 50 | -0.002 | -0.008 – 0.005 | -0.025 – 0.011 |
| | ≥75 | 1 | -10.915 | -11.0 – -10.8 | -11.1 – -10.7 |
| | | 5 | -3.703 | -3.75 – -3.66 | -3.89 – -3.52 |
| | | 30 | -0.190 | -0.204 – -0.178 | -0.223 – -0.156 |
| | | 50 | -0.026 | -0.030 – -0.020 | -0.041 – -0.010 |
| **RSF-ISR** | All | 1 | 0.102 | 0.099 – 0.106 | 0.088 – 0.116 |
| | | 5 | 0.032 | 0.030 – 0.034 | 0.026 – 0.040 |
| | | 30 | 0.023 | 0.020 – 0.028 | 0.014 – 0.034 |
| | | 50 | 0.027 | 0.024 – 0.030 | 0.019 – 0.037 |
| | ≥75 | 1 | 0.159 | 0.156 – 0.161 | 0.148 – 0.167 |
| | | 5 | 0.062 | 0.060 – 0.064 | 0.056 – 0.070 |
| | | 30 | 0.038 | 0.037 – 0.040 | 0.032 – 0.046 |
| | | 50 | 0.037 | 0.035 – 0.039 | 0.030 – 0.045 |
| **RSF-Dykstra** | All | 1 | -13.067 | -13.2 – -12.9 | -13.4 – -12.6 |
| | | 5 | -3.072 | -3.15 – -2.99 | -3.32 – -2.85 |
| | | 30 | -0.098 | -0.123 – -0.082 | -0.150 – -0.063 |
| | | 50 | -0.003 | -0.010 – 0.003 | -0.026 – 0.010 |
| | ≥75 | 1 | -14.653 | -14.8 – -14.5 | -15.0 – -14.4 |
| | | 5 | -4.015 | -4.07 – -3.97 | -4.22 – -3.83 |

| Model (Calibration) | Training data | Case-control Ratio (control / case) | Median | IQR | 95% bootstrap CI |
|---|---|---|---|---|---|
| | | 30 | -0.201 | -0.215 – -0.188 | -0.235 – -0.166 |
| | | 50 | -0.028 | -0.032 – -0.021 | -0.043 – -0.012 |
| **RSF-ISR-Dykstra** | All | 1 | -0.730 | -0.785 – -0.669 | -0.923 – -0.584 |
| | | 5 | 0.003 | -0.003 – 0.008 | -0.013 – 0.019 |
| | | 30 | 0.016 | 0.011 – 0.022 | 0.004 – 0.030 |
| | | 50 | 0.022 | 0.017 – 0.027 | 0.010 – 0.034 |
| | ≥75 | 1 | -1.521 | -1.614 – -1.439 | -1.821 – -1.332 |
| | | 5 | 0.022 | 0.015 – 0.028 | 0.006 – 0.037 |
| | | 30 | 0.025 | 0.021 – 0.029 | 0.014 – 0.037 |
| | | 50 | 0.028 | 0.025 – 0.032 | 0.018 – 0.039 |

*Note:* Values represent median, interquartile range (IQR, 25th–75th percentiles), and 95% bootstrap confidence interval across 100 ID-level bootstrap iterations. Negative IBSS reflects performance inferior to the population Kaplan–Meier benchmark. CI: confidence interval; IBSS: integrated Brier skill score; IQR: interquartile range; RSF: random survival forest; RW-ISR: reweighted isotonic survival regression.

**Supplementary Table S4. Predictive performance of benchmark Cox proportional hazards models among individuals aged ≥75 years.**

| Feature Set | Ratio | Median C-index (95% CI) | Uncalibrated IBSS (95% CI) | RW-ISR Calibrated IBSS (95% CI) |
|---|---|---|---|---|
| **Demographic Cox** (Age, Sex) | 1:1 | 0.707 (0.707 – 0.707) | -14.947 (-15.137 – -14.739) | 0.030 (0.026 – 0.034) |
| | 1:5 | 0.728 (0.728 – 0.728) | -3.980 (-4.203 – -3.795) | 0.032 (0.028 – 0.036) |
| | 1:30 | 0.728 (0.727 – 0.728) | -0.235 (-0.267 – -0.194) | 0.032 (0.028 – 0.037) |
| | 1:50 | 0.728 (0.727 – 0.728) | -0.034 (-0.055 – -0.020) | 0.032 (0.028 – 0.037) |
| **Clinical Cox** (Age, Sex, 46 Checkups) | 1:1 | 0.674 (0.670 – 0.678) | -8.489 (-8.701 – -8.280) | 0.214 (0.208 – 0.219) |
| | 1:5 | 0.756 (0.753 – 0.760) | -2.849 (-3.017 – -2.689) | 0.092 (0.086 – 0.099) |
| | 1:30 | 0.770 (0.766 – 0.775) | -0.202 (-0.246 – -0.157) | 0.047 (0.042 – 0.054) |
| | 1:50 | 0.769 (0.762 – 0.774) | -0.028 (-0.053 – 0.000) | 0.042 (0.035 – 0.052) |
| **All-Features Cox** (All 488 Predictors) | 1:1 | 0.690 (0.686 – 0.695) | -8.239 (-8.443 – -8.037) | 0.187 (0.178 – 0.199) |
| | 1:5 | 0.764 (0.759 – 0.770) | -2.767 (-2.942 – -2.613) | 0.088 (0.080 – 0.096) |
| | 1:30 | 0.764 (0.754 – 0.772) | -0.247 (-0.298 – -0.196) | 0.043 (0.033 – 0.051) |
| | 1:50 | 0.752 (0.740 – 0.763) | -0.078 (-0.106 – -0.044) | 0.036 (0.027 – 0.044) |

*Note:* Values represent median (95% bootstrap confidence interval) across 100 ID-level bootstrap repetitions evaluated on the independent evaluation dataset (aged ≥75 years). All Cox models were regularized using an elastic net penalty (mixing parameter ℓ1 = 0.5) with standard feature scaling. CI: confidence interval; IBSS: integrated Brier skill score; RW-ISR: reweighted isotonic survival regression.

**Supplementary Table S5. Multi-horizon calibration performance metrics for uncalibrated and calibrated RSF models at 1, 3, and 5 years.**

| Follow-up Horizon | Calibration Metric | Uncalibrated RSF (1:1 ratio) (95% CI) | Calibrated RSF-ISR (1:1 ratio) (95% CI) |
|---|---|---|---|
| **1-Year (12 Months)** | Calibration Slope | 1.92 (1.83 – 2.01) | 0.91 (0.86 – 0.95) |
| | Calibration-in-the-Large (CIL) | -3.95 (-3.97 – -3.92) | -0.12 (-0.14 – -0.10) |
| | Observed-to-Expected Ratio (O:E) | 0.025 (0.025 – 0.026) | 0.89 (0.88 – 0.90) |
| **3-Year (36 Months)** | Calibration Slope | 2.57 (2.45 – 2.67) | 0.90 (0.87 – 0.92) |
| | Calibration-in-the-Large (CIL) | -3.87 (-3.89 – -3.84) | -0.12 (-0.13 – -0.10) |
| | Observed-to-Expected Ratio (O:E) | 0.043 (0.043 – 0.044) | 0.91 (0.90 – 0.92) |
| **5-Year (60 Months)** | Calibration Slope | 3.06 (2.89 – 3.25) | 0.75 (0.65 – 0.84) |
| | Calibration-in-the-Large (CIL) | -4.07 (-4.11 – -4.03) | -0.04 (-0.06 – -0.02) |
| | Observed-to-Expected Ratio (O:E) | 0.057 (0.056 – 0.057) | 0.97 (0.96 – 0.98) |

*Note:* Evaluated on the independent evaluation dataset restricted to individuals aged ≥75 years (tra75 cohort, 1:1 case-control training ratio) across 100 bootstrap repetitions. Values represent median (95% bootstrap confidence interval). CI: confidence interval; RSF: random survival forest; RW-ISR: reweighted isotonic survival regression.

**Supplementary Table S6. Sensitivity analysis evaluating discrimination and calibration using single health checkup records per individual.**

| Model Strategy | Sampling Ratio | First Checkup: C-index (95% CI) | First Checkup: IBSS (95% CI) | Latest Checkup: C-index (95% CI) | Latest Checkup: IBSS (95% CI) |
|---|---|---|---|---|---|
| **RSF (Uncalibrated)** | 1:1 | 0.691 (0.678 – 0.703) | -17.58 (-19.71 – -15.82) | 0.761 (0.751 – 0.772) | -16.89 (-18.95 – -15.11) |
| | 1:5 | 0.739 (0.727 – 0.751) | -2.30 (-2.61 – -1.96) | 0.752 (0.739 – 0.764) | -2.38 (-2.70 – -2.04) |
| | 1:30 | 0.753 (0.738 – 0.768) | -0.016 (-0.030 – -0.005) | 0.767 (0.752 – 0.781) | -0.021 (-0.037 – -0.009) |
| | 1:50 | 0.751 (0.736 – 0.766) | 0.052 (0.042 – 0.061) | 0.765 (0.750 – 0.779) | 0.049 (0.038 – 0.059) |
| **RSF-ISR (Calibrated)** | 1:1 | 0.691 (0.678 – 0.703) | 0.147 (0.136 – 0.158) | 0.761 (0.751 – 0.772) | 0.154 (0.143 – 0.165) |
| | 1:5 | 0.739 (0.727 – 0.751) | 0.114 (0.099 – 0.127) | 0.752 (0.739 – 0.764) | 0.108 (0.093 – 0.122) |
| | 1:30 | 0.753 (0.738 – 0.768) | 0.078 (0.064 – 0.092) | 0.767 (0.752 – 0.781) | 0.074 (0.059 – 0.088) |
| | 1:50 | 0.751 (0.736 – 0.766) | 0.079 (0.066 – 0.092) | 0.765 (0.750 – 0.779) | 0.075 (0.061 – 0.088) |

*Note:* Values represent median (95% bootstrap confidence interval) across 100 ID-level bootstrap repetitions evaluated on independent evaluation datasets restricted strictly to a single checkup record per person (aged ≥75 years). CI: confidence interval; IBSS: integrated Brier skill score; RSF: random survival forest; RW-ISR: reweighted isotonic survival regression.

**Supplementary Table S7. Temporal monotonicity evaluation of individual predicted survival curves across follow-up time points.**

| Metric | RSF (Uncalibrated) | RW-ISR (Calibrated) | Dykstra Projection | RW-ISR-Dykstra |
|---|---|---|---|---|
| **Strictly Monotonic Curves (%)** | 100.0% (100.0 – 100.0%) | 96.1% (95.4 – 96.7%) | 100.0% (100.0 – 100.0%) | 100.0% (100.0 – 100.0%) |
| **Non-Monotonic Intervals (%)** | 0.0% (0.0 – 0.0%) | 3.9% (3.3 – 4.6%) | 0.0% (0.0 – 0.0%) | 0.0% (0.0 – 0.0%) |
| **Mean Maximum Reversal (ΔS)** | 0.000 | 0.089 (0.078 – 0.101) | 0.000 | 0.000 |
| **IBSS at 1:1 Ratio** | -17.636 | **0.159** | -1.521 | 0.061 |
| **Calibration Slope (1-yr / 5-yr)** | 1.92 / 3.06 | **0.91 / 0.75** | 0.42 / 0.45 | 0.58 / 0.60 |

*Note:* Evaluated across 71 monthly follow-up points on the independent evaluation dataset (aged ≥75 years, 1:1 ratio) over 100 bootstrap iterations. Non-monotonic interval rate denotes proportion of adjacent monthly points where $S(t+1) > S(t)$. Values represent median (95% bootstrap confidence interval). CI: confidence interval; IBSS: integrated Brier skill score; RSF: random survival forest; RW-ISR: reweighted isotonic survival regression.
